# Association of three putative periodontal pathogens with chronic periodontitis in Brazilian subjects

**DOI:** 10.1590/1678-775720150445

**Published:** 2016

**Authors:** Cristiane GONÇALVES, Geisla Mary S. SOARES, Marcelo FAVERI, Paula Juliana PÉREZ-CHAPARRO, Eduardo LOBÃO, Luciene Cristina FIGUEIREDO, Gustavo Titonele BACCELLI, Magda FERES

**Affiliations:** Universidade de Guarulhos, Departamento de Periodontia, Guarulhos, SP, Brasil.

**Keywords:** Chronic periodontitis, Etiology, Pathogens, Microbiology

## Abstract

**Objective:**

The aim of this study was to evaluate the association of *Porphyromonas endodontalis*, *Filifactor alocis* and *Dialister pneumosintes* with the occurrence of periodontitis.

**Material and Methods:**

Thirty subjects with chronic periodontitis (ChP) and 10 with periodontal health (PH) were included in the study. Nine subgingival biofilm samples were collected as follows: i) PH group - from the mesial/buccal aspect of each tooth in two randomly chosen contralateral quadrants; ii) ChP group - from three sites in each of the following probing depth (PD) categories: shallow (≤3 mm), moderate (4-6 mm) and deep (≥7 mm). Checkerboard DNA-DNA hybridization was used to analyze the samples.

**Results:**

We found the three species evaluated in a higher percentage of sites and at higher levels in the group with ChP than in the PH group (p<0.05, Mann-Whitney test). We also observed these differences when the samples from sites with PD≤4 mm or ≥5 mm of subjects with ChP were compared with those from subjects with PH (p<0.05, Mann-Whitney test). In addition, the prevalence and levels of *D. pneumosintes*, and especially of *F. alocis* were very low in healthy subjects (0.12x10^5^ and 0.01x10^5^, respectively).

**Conclusion:**

*F. alocis* and *D. pneumosintes* might be associated with the etiology of ChP, and their role in the onset and progression of this infection should be further investigated. The role of *P. endodontalis* was less evident, since this species was found in relatively high levels and prevalence in the PH group.

## INTRODUCTION

The oral cavity naturally hosts hundreds of species that together constitute the oral microbiome. Most of these microorganisms are compatible with a good oral health status, while some of them are considered pathogens that might trigger infectious processes, such as periodontitis. Knowledge about the periodontal pathogens associated with the onset and progression of periodontal diseases has been largely concentrated on the microorganisms that comprise the “subgingival microbial complexes” previously described by Socransky, et al.^24^ ^,^ ^25^ (2002, 1998). Nonetheless, a recent systematic review has indicated the existence of other candidate periodontal pathogens^20^.

The first line of evidence to define a microorganism as a true pathogen is to determine if the organism is present in a higher prevalence and/or levels and/or proportions/abundance in disease than in health (association studies)^22^. The increased levels of a bacterial species during disease is one of the most important parameters to evaluate its role as a periodontal pathogen, and is more meaningful than the mere presence or absence of the microorganism in the subgingival environment^22^ ^,^ ^24^. Some investigations have shown that *Porphyromonas endodontalis, Filifactor alocis* and *Dialister pneumosintes* are present in a higher prevalence and/or abundance in patients with periodontitis than in periodontally healthy subjects^1^ ^,^ ^2^ ^,^ ^5^ ^,^ ^7^ ^,^ ^10^ ^,^ ^14^ ^,^ ^15^ ^,^ ^18^ ^,^ ^21^. However, to our knowledge, no studies to date have compared the prevalence as well as the levels of these three species in subjects with good periodontal health and with disease.

Therefore, the aim of this study was to evaluate the association of three putative periodontal pathogens, *P. endodontalis, F. alocis* and *D. pneumosintes* with periodontitis, by comparing their levels and prevalence in subjects with chronic periodontitis (ChP) and those in good periodontal health (PH). This knowledge might help to establish more effective preventive and treatment strategies for these infections.

## MATERIAL AND METHODS

### Study population

Ten periodontally healthy subjects and 30 subjects with ChP were selected from the population referred to the Periodontal Clinic of Guarulhos University (Guarulhos, SP, Brazil) for treatment. Detailed medical and dental records were obtained, and one trained and calibrated examiner performed a full-mouth periodontal examination. Subjects who fulfilled the inclusion criteria were invited to participate in the study. All eligible subjects were informed of the nature, potential risks and benefits of their participation in the study and signed an informed consent document. The Guarulhos University’s Clinical Research Ethics Committee approved the study protocol.

### Inclusion and exclusion criteria

Inclusion criteria for periodontally healthy subjects were: >30 years, ≥24 teeth, no sites with probing depth (PD) and/or clinical attachment level (CAL) ≥3 mm and fewer than 20% of sites with gingival bleeding and/or bleeding on probing (BOP). Inclusion criteria for subjects with periodontitis were: >30 years, ≥20 teeth, ≥8 sites in different teeth with PD≥5 mm, CAL≥3 mm and BOP.

Exclusion criteria were pregnancy, lactation, currently being a smoker, antimicrobial therapies during the previous 6 months, medical conditions requiring prophylactic antibiotic coverage, continuous use of mouth-rinses containing antimicrobial agents in the preceding 3 months, systemic conditions that could affect the progression of periodontitis and long-term administration of anti-inflammatory and immunosuppressive medications.

### Clinical evaluation

One examiner performed the clinical monitoring and carried out all clinical measurements in a given subject. Visible plaque (0/1), gingival bleeding (0/1), BOP (0/1), suppuration (0/1), PD and CAL were measured at six sites per tooth (mesiobuccal, buccal, distobuccal, distolingual, lingual and mesiolingual) in all teeth, excluding third molars. PD and CAL measurements were recorded to the nearest millimeter using a North Carolina periodontal probe (Hu-Friedy, Chicago, IL, USA).

### Microbiological evaluation

#### Sample collection

After having recorded the clinical parameters, supragingival plaque was removed and then nine individual subgingival plaque samples were collected per subject using sterile 11/12 mini-Gracey curettes as follows: Periodontally healthy subjects: samples were collected from the mesial/buccal aspect of each tooth in two randomly chosen contralateral quadrants. Subjects with ChP: samples were collected from three sites in each of the following PD categories: shallow (PD≤3 mm), moderate (PD 4-6 mm) and deep (PD≥7 mm).

The samples were placed in separate plastic tubes containing 0.15 mL of TE (10 mM Tris- HCl, 1mM EDTA, pH 7.6). One hundred microliters of 0.5 M NaOH were immediately added to each tube and the samples were stored at -80°C.

#### Checkerboard DNA-DNA hybridization

The samples were evaluated by checkerboard DNA–DNA hybridization^19^ ^,^ ^23^ at the Guarulhos University Laboratory of Microbiology. The samples were boiled for 10 min and 0.8 ml of 5 M ammonium acetate was added to each sample. The DNA released was then placed into the extended slots of a Minislot 30 apparatus (Immunetics, Cambridge, MA, USA), concentrated on a 15/15 cm, positively charged, nylon membrane (Boehringer Mannheim, Indianapolis, IN, USA) and fixed to the membrane by baking it at 120°C for 20min. The membrane was then placed in a Miniblotter 45 (Immunetics) with the lanes of DNA at 90°C in the lanes of the device. Digoxigenin-labelled whole genomic DNA probes for *P. endodontalis*, *F. alocis and D. pneumosintes* were hybridized in individual lanes of the Miniblotter. After hybridization, the membranes were washed at high stringency and the DNA probes were detected using the antibody to digoxigenin conjugated with alkaline phosphatase, and chemiluminescence detection. The last two lanes in each run contained standards at concentrations of 10^5^ and 10^6^ cells of each species. Signals were evaluated by comparison with the standards at 10^5^ and 10^6^ bacterial cells for the test species on the same membrane. The sensitivity of this assay was adjusted to allow detection of each DNA probe. This procedure was carried out in order to provide the same sensitivity of detection for each of the species.

## Statistical analysis

Each individual clinical parameter as well as mean counts (x10^5^) of each bacterial species evaluated and the percentage of sites colonized by these species were computed per subject and then across subjects in both groups. The Mann–Whitney test was used to seek significance of differences between the two groups for age, clinical and microbiological parameters, and applying Bonferroni’s correction for multiple comparisons. Thus, from this computation, a p value of <0.016 was considered to be statistically significant at p<0.05. Similarly, individual values of p<0.003 and p<0.0003 were considered statistically significant at p<0.01 and p<0.001, respectively^26^. A Chi-square test was used to compare differences in the frequency of gender. Levels of the three bacterial species were averaged separately within two PD categories (PD ≥5 mm and PD ≤4 mm) per subject, and then across subjects with ChP. The Wilcoxon Test was used to find the significance of differences between these two categories. The level of significance was set at 5%.

## RESULTS

Table 1 presents the demographic and clinical characteristics of the subjects with ChP and PH. As expected, we observed statistically significant differences between the two groups for all clinical parameters evaluated (p<0.05). The mean age and percentage of females did not differ between groups (p>0.05).

**Table 1:** Clinical parameters and demographic characteristics of the two groups

	Healthy	Periodontitis	
	(n=10)	(n=30)	Mann-Whitney
	Mean±SD	Mean±SD	(p)
Number of females	9	22	0.73889
Age	38.3±6.8	42.2±6.4	0.26
PD	1.8±0.2	3.7±0.7	0.0
CAL	0.9±0.2	4.5±1.3	0.0
% sites with:			
Plaque	25.5±10.1	80.0±15.6	0.0
Gingival bleeding	2.0±0.8	27.7±15.3	0.0
Bleeding on probing	3.1±1.2	78.9±14.1	0.0
Suppuration	0.0±0.0	1.1±0.8	0.00001

PD: probing depth; CAL: clinical attachment level; SD: standard deviation

**Figure 1:**
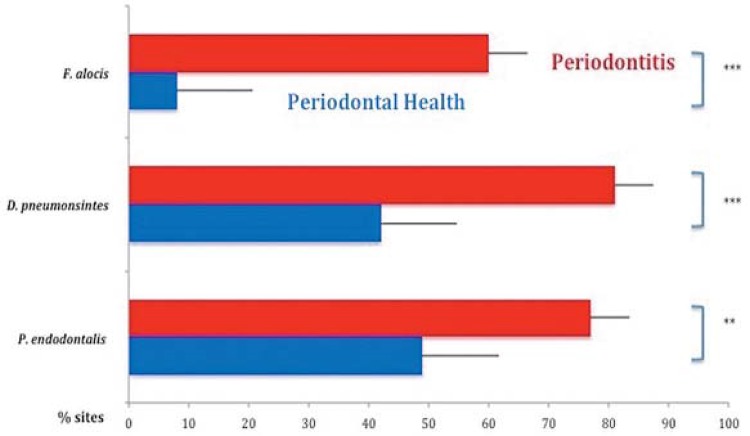
Mean percentage of sites colonized by the three bacterial species evaluated. The Mann-Whitney test was used to assess significance of differences between groups; **p<0.01 and ***p<0.001

**Figure 2:**
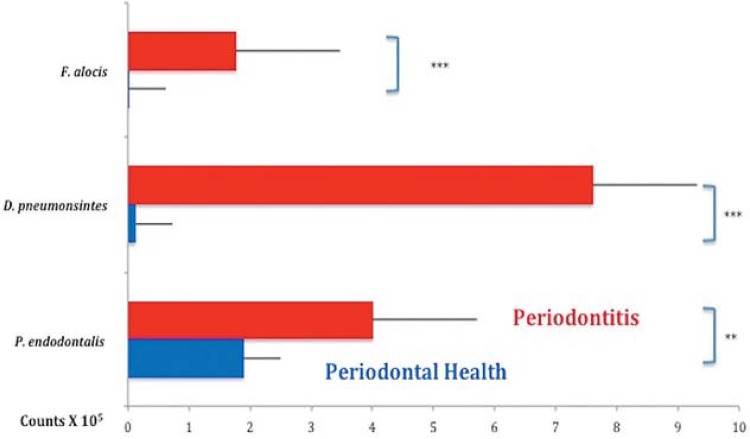
Mean counts (x105) of the three bacterial species evaluated. The Mann-Whitney test was used to assess significance of differences between groups; **p<0.01 and ***p<0.001

**Figure 3:**
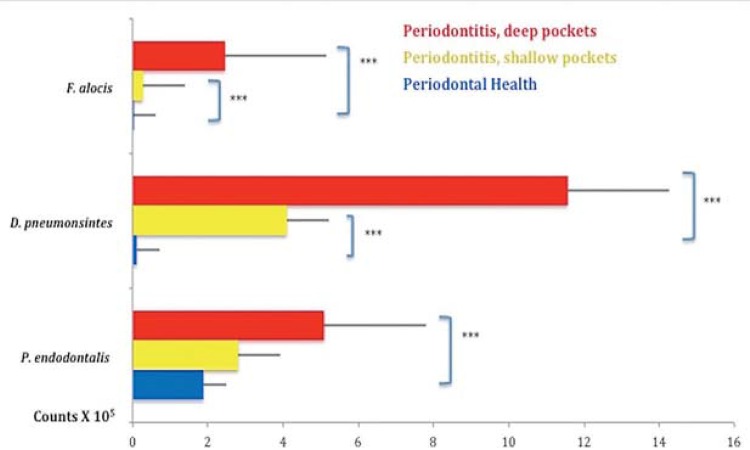
Mean counts (x105) of the three bacterial species evaluated in periodontally health and in sites with probing depth ≥5 mm and ≤4 mm in subjects with periodontitis. The Mann-Whitney test; was used to assess the significance of differences between the healthy group and each of the PD category subgroup; ***p<0.001

Figures 1 and 2 present the mean percentage of sites colonized by *F. alocis*, *D. pneumosintes* and *P. endodontalis* and the mean counts of these species, respectively, in the two clinical groups. All three species were present in a statistically significant, higher percentage of sites and mean counts in subjects with ChP than in those in PH (p<0.05). The mean levels of *F. alocis*, *D. pneumosintes* and *P. endodontalis* in the healthy subjects were 0.01x10^5^, 0.12x10^5^ and 1.89x10^5^, respectively; and 1.77x10^5^, 7.61x10^5^ and 4.01x10^5^, respectively, in the periodontitis subjects. Figure 3 shows the mean counts (x10^5^) of the three microbial species in subjects with PH and in sites with PD ≥5 mm and ≤4 mm in subjects with ChP. *F. alocis* and *D. pneumosintes* were present in statistically significant, higher mean levels in shallow and deep sites of periodontitis subjects in comparison with the levels observed in healthy subjects (p<0.05). The levels of *P. endodontalis* did not differ significantly between shallow sites of subjects with ChP and PH.

## DISCUSSION

We found the three candidate periodontal pathogens evaluated in this study in statistically significant higher percentage of sites and in higher levels in subjects with ChP than in periodontally healthy individuals. This data is in agreement with previous investigations that also showed an association of *P. endodontalis* ^1^ ^,^ ^6^ ^,^ ^10^ ^,^ ^15^ ^-^ ^17^ *, F. alocis* ^1^ ^-^ ^3^ ^,^ ^6^ ^,^ ^8^ ^,^ ^10^ ^,^ ^14^ ^,^ ^15^ ^,^ ^21^ and *D. pneumosintes* ^7^ ^,^ ^8^ ^,^ ^14^ ^,^ ^18^ with periodontal diseases. In addition, a recent Systematic Review, which evaluated the weight of evidence for the existence of novel periodontal pathogens, suggested that 32 newly identified taxa might be associated with the etiology of periodontitis. The authors proposed that there is moderate evidence in the literature to support the role of *P. endodontalis* and *F. alocis* as periodontal pathogens, and some evidence for *D. pneumosintes* ^20^.

Although levels of the three bacterial species evaluated in this study were elevated in subjects with ChP in comparison with periodontally healthy individuals (Figures 1 and 2), their levels and prevalence in healthy subjects also provided essential information as regards their possible role in the etiology of the disease. *F. alocis* was present in only 8% of the sites of healthy individuals and at insignificant levels. *D. pneumosintes* was also present at very low levels in healthy individuals, but was detected in 42% of the sites evaluated. We found *P. endodontalis* in almost 50% of the sites of healthy subjects and at relatively high levels. This data might indicate that *P. endodontalis* is an opportunistic bacterial species or an accessory pathogen that may not trigger the disease process, but whose levels may increase when the inflammation process begins, thus contributing to the disease process^12^. Apparently, *F. alocis* retains the characteristics of a so called “keystone pathogen”, microorganisms that have the potential to initiate periodontal destruction by causing a dysbiosis in the subgingival ecosystem^3^ ^,^ ^13^. Although its (*F. alocis)* prevalence and levels in healthy subjects was very low in this study, Aruni, et al.^3^ ^,^ ^4^ (2014,2011) showed that *F. alocis* has some unique virulence factors that favor its persistence in inflammatory environments and may mediate *P. gingivalis* proliferation, suggesting that this species can play a pivotal role in microbial community dynamics.

The strength of this study was the number of subgingival plaque samples analyzed for the levels and prevalence of the three bacterial species. Although the number of subjects included in this study was relatively small, 360 plaque samples were individually analyzed. To date, most of the association studies that have investigated *F. alocis*, *D. pneumosintes* or *P. endodontalis* evaluated fewer samples^1^ ^,^ ^6^ ^,^ ^9^ ^,^ ^10^ ^,^ ^15^ ^,^ ^16^ ^,^ ^18^ ^,^ ^21^. In addition, these studies have generally only determined the prevalence or abundance (proportion) of these microbial species in the subgingival biofilm. Indeed, studies have recognized that evaluating a large number of plaque samples and quantifying the microorganisms are critical requisites when trying to establish an association between certain microorganisms and the onset and progression of periodontitis^11^.

It is important to mention that association studies only provide the initial information necessary to suggest a possible link between a microorganism and an infection. Further evaluations are necessary to confirm this type of association, such as clinical (i.e. risk assessment and interventional studies); host-response studies, and investigations into the mechanisms of pathogenicity of the suspected pathogens.

## CONCLUSION

In conclusion, the results of this investigation suggested an association of *D. pneumosintes*, and especially of *F. alocis* with the etiology of periodontitis. The role of *P. endodontalis* was less evident, since we found this species in relatively high levels and prevalence in periodontally healthy subjects. This data might guide future studies on the actual role of these three bacterial species in the etiology of periodontitis and help to establish a more effective treatment for these infections.
